# Fighting Against Promoter DNA Hyper-Methylation: Protective Histone Modification Profiles of Stress-Resistant Intestinal Stem Cells

**DOI:** 10.3390/ijms21061941

**Published:** 2020-03-12

**Authors:** Torsten Thalheim, Lydia Hopp, Maria Herberg, Susann Siebert, Christiane Kerner, Marianne Quaas, Michal R. Schweiger, Gabriela Aust, Joerg Galle

**Affiliations:** 1 Interdisciplinary Center for Bioinformatics (IZBI), Leipzig University, 04107 Leipzig, Germany; thalheim@izbi.uni-leipzig.de (T.T.); hopp@izbi.uni-leipzig.de (L.H.); herberg@izbi.uni-leipzig.de (M.H.); 2 Laboratory for Translational Epigenetics and Tumor Genetics, University Hospital Cologne, 50391 Cologne, Germany; susann.siebert@uk-koeln.de (S.S.); mschweig@uni-koeln.de (M.R.S.); 3 Center for Molecular Medicine Cologne, CMMC, 50391 Cologne, Germany; 4 Department of Surgery, Research Laboratories, Leipzig University, 04103 Leipzig, Germany; Christiane.Kerner@medizin.uni-leipzig.de (C.K.); Marianne.Quaas@medizin.uni-leipzig.de (M.Q.); Gabriela.Aust@medizin.uni-leipzig.de (G.A.)

**Keywords:** promoter DNA hyper-methylation, histone methylation, DNA damage, irradiation, loss of Msh2 function, computational modeling

## Abstract

Aberrant DNA methylation in stem cells is a hallmark of aging and tumor development. Recently, we have suggested that promoter DNA hyper-methylation originates in DNA repair and that even successful DNA repair might confer this kind of epigenetic long-term change. Here, we ask for interrelations between promoter DNA methylation and histone modification changes observed in the intestine weeks after irradiation and/or following *Msh2* loss. We focus on H3K4me3 recruitment to the promoter of H3K27me3 target genes. By RNA- and histone ChIP-sequencing, we demonstrate that this recruitment occurs without changes of the average gene transcription and does not involve H3K9me3. Applying a mathematical model of epigenetic regulation of transcription, we show that the recruitment can be explained by stronger DNA binding of H3K4me3 and H3K27me3 histone methyl-transferases as a consequence of lower DNA methylation. This scenario implicates stable transcription despite of H3K4me3 recruitment, in agreement with our RNA-seq data. Following several kinds of stress, including moderate irradiation, stress-sensitive intestinal stem cell (ISCs) are known to become replaced by more resistant populations. Our simulation results suggest that the stress-resistant ISCs are largely protected against promoter hyper-methylation of H3K27me3 target genes.

## 1. Introduction

Our epigenome is subject to age-related changes, among them are alterations of DNA methylation. Two antagonistic trends have been identified: an overall DNA hypo-methylation and a hyper-methylation of a subgroup of gene promoters [1]. While these changes are common and used to predict biological age [2,3,4], the mechanisms behind these changes are still not fully understood. The genes affected by promoter DNA hyper-methylation are mostly polycomb target genes [5,6]. Accordingly, the nucleosomes, which are associated with them, carry a tri-methylation at lysine 27 of histone H3 (H3K27me3). This modification is associated with gene silencing [7] and, consistently, most of the modified genes are not transcribed. Functional annotation of them demonstrated enrichment in developmental genes [8].

DNA damage is triggered either endogenously by metabolism or exogenously by environmental factors. It occurs thousands of times on average per day per cell requiring permanent DNA repair [9]. Applying a multi-scale computer model of epigenetic regulation of transcription, we have demonstrated that promoter DNA hyper-methylation is a potential consequence of DNA repair [10]. In the model, we considered that DNA repair is associated with chromatin opening and improved recruitment of *de novo* DNA methyltransferases (DNMTs) [11,12]. Moreover, we assumed histone state-dependent recruitment of *de novo* DNMTs and methylation-dependent binding of maintenance DNMTs to CpGs, in accordance with experimental findings [13,14,15,16]. Our results suggest that DNA repair facilitates promoter hyper-methylation and that methylation-dependent DNA binding of the maintenance DNMT, DNMT1, is a prerequisite of this process. In experiments and simulations, promoter hyper-methylation was most frequent at H3K27me3 target genes and not seen for genes associated with nucleosomes that carry high levels of tri-methylation at lysine 4 of histone H3 (H3K4me3).

DNA damage increases after loss of function of DNA repair mechanisms, as frequently seen in tumors [17]. Thus, in agreement with the above assumptions, promoter DNA hyper-methylation is enforced during tumor formation [6]. Similarly, one would expect that this kind of epigenetic drift is accelerated in individuals that are continuously exposed to higher doses of radiation as well. Indeed, increased promoter DNA methylation was reported in nuclear industry workers [18,19].

Recently, we have analyzed genome-wide histone H3 methylation profiles in macroscopic normal intestinal tissue of irradiated and untreated mismatch-repair-deficient *VC^+/?^Msh2^LoxP/LoxP^ (Msh2^−/−^)* mice [20]. We observed common long-term stable changes in histone H3 methylation following irradiation and Msh2 loss. Among the genes that underwent such changes, we identified genes that are H3K27me3 targets in control mice (*Msh2*^+/+^). A subgroup of them recruited H3K4me3 to histones associated with their promoter. The measurements were performed on the population level. Thus, the recruitment either indicates a switch from H3K27me3 to H3K4me3 modification of these genes in a subgroup of cells, or is associated with the induction of actually bivalent modification. Affected genes enrich in gene sets typically enriched with H3K27me3 targets [20]. However, their promoters have a lower average DNA methylation level in control mice compared with other H3K27me3 target genes, as observed utilizing data published by Kazakevych et al. [21]. Questions are raised on the function of this recruitment.

On the basis of RNA-seq data, we demonstrate that the observed H3K4 modification changes are not paralleled by changes in the average transcription. Applying *in silico* simulation, we show that a potential origin of the H3K4me3 recruitment is low *de novo* DNA methylation activity. Changes of the steady-state distribution of epigenetic states caused by this low activity explain why, despite of H3K4me3 recruitment, gene transcription remains stable. We predict that promoters of affected genes are protected against hyper-methylation in the course of DNA repair and discuss DNA damage response scenarios that enable such regulation.

## 2. Results

### 2.1. Experimental Results

#### 2.1.1. Definition of G1 Genes by Histone Modification Profiles

In our study, we focus on H3K27me3 target genes that were observed to recruit H3K4me3 after irradiation and/or loss of *Msh2* in the intestine, that is, under genomic stress [20]. To complement H3K4me3, H3K27me3, and H3K36me3 profiles provided by Herberg et al. [20], we performed ChIP-seq measurement for H3K9me3 in macroscopic normal intestinal tissue of the same irradiated (rad) and untreated Msh2^+/+^ and Msh2^−/−^ mice. This repressive histone modification was shown to control lineage specification of stem cells in cooperation with H3K4me3, similar to H3K27me3 [22]. In contrast to H3K4me3, H3K27me3, and H3K36me3, only a small fraction (<25%) of the H3K9me3 peaks are associated with genes independent of the mouse genotype and radiation status (Supplementary Figure S1). We assigned all genes a histone modification state, which is encoded as 0/1-quadruplet: (H3K4me3, H3K9me3, H3K27me3, H3K36me3). 

**Figure 1:**
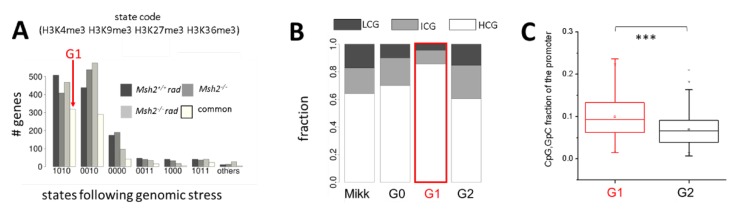
Properties of G1 genes. (**A**) Histone modification state changes of genes exclusively modified by H3K27me3 (0010) in *Msh2^+/+^* mice following genomic stress. The H3K27me3 targets frequently recruit H3K4me3. Gene set G1 comprises those (0010) genes that show common H3K4m3 recruitment in all mice. (**B**) Genome-wide, about two-thirds of all genes are high CpG (HCG) promoter genes, that is, genes with promoters with high CpG density (Mikk: Mikkelsen et al. [23]). Promotors of G1 genes enrich in HCG promoter genes, while G2 genes comprise higher fractions of intermediate CpG (ICG) and low CpG (LCG) promoter genes compared with the set of all (0010) genes in *Msh2^+/+^* mice (set G0). (**C**) Boxplot of the CpG, GpC fraction of the promoters of G1 and G2 genes demonstrating significant differences (*** *p*-value < 1e-11, Kolmogorov–Smirnov test).

Among all genes that recruit H3K4me3 under genomic stress, we found (0010) H3K27me3 targets to be most efficient. A large fraction of these genes consistently recruit H3K4me3 under different types of genomic stress (Figure 1A). Thus, H3K4me3 recruitment is a common feature of (0010) H3K27me3 targets in the intestine under genomic stress.

In the following, we call the set of those genes, which changed their state from (0010) in *Msh2^+/+^* mice to (1010) in *Msh2^+/+^* rad, *Msh2^−/−^*, and *Msh2^−/−^* rad mice, ‘G1′ (319 genes). All (0010) genes that never recruited H3K4me3 are used as a control set. We call this gene set ‘G2′ (557 genes).

#### 2.1.2. H3K4me3 Recruitment after Irradiation and/or Loss of Msh2 Is a Feature of High CpG Promoters

G1 genes show low promoter DNA methylation in control mice [20]. As DNA methylation depends on the local density of CpGs [23], we hypothesized that this feature of G1 genes refers to their promoter type. We utilized experimental data provided by Mikkelsen et al. [23] that classify promoters into high (HCG), intermediate (ICG), and low CpG density (LCG) promoters. Consistent with their low methylation level, we found the promoters of G1 genes to be enriched in HCG promoter genes (Figure 1B). Their CpG, GpC fraction is significantly higher compared with that of G2 genes (Figure 1C).

#### 2.1.3. H3K4me3 Recruitment by G1 Genes after Irradiation and/or Loss of Msh2 Does not Change Their Transcription

**Figure 2:**
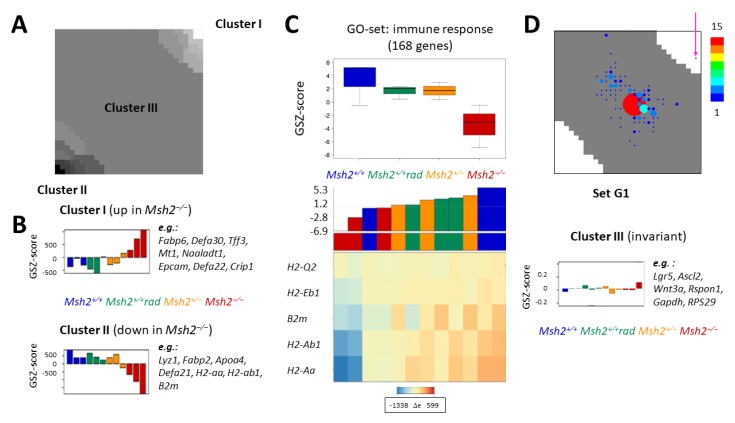
Gene transcription under genomic stress. Results of a self-organizing map (SOM) analysis of RNA-seq data. (**A**) Gene clusters (different grey values) obtained based on correlated gene transcription across all analyzed intestinal samples. (**B**) The gene set enrichment Z- (GSZ) scores of the genes of clusters I (white in A) and II (black in A) are shown for all samples analyzed. Representative genes of the clusters are indicated. (**C**) Transcription of the genes of the gene ontology (GO) set ‘Immune response’ demonstrating their weak down-regulation in *Msh2^+/+^* rad and *Msh2^+/−^* mice and strong repression in *Msh2^−/−^* mice. Group GSZ-score (upper panel), sample GSZ-score (middle panel), and relative expression Δe (RPKM) of the five most pronounced regulated genes of the set (lower panel). The distribution of the entire set within the SOM is given in Figure S2C. (**D**) Characteristics of G1 gene transcription. Except for one (pink arrow), all G1 genes are part of the cluster III of invariant transcription (grey). Color scale: number of genes per metagene. GSZ-scores of all cluster III genes across all samples are shown below.

In order to further characterize G1 genes, we applied RNA-seq analysis in untreated *Msh2*^+/+^, *Msh2^+/−^*, *Msh2*^−/−^, and irradiated *Msh2*^+/+^ mice (for RT-PCR validation, see Supplementary I, Figure S2A). Histone profiles specifying the G1 set were obtained four weeks after irradiation, indicating long term changes in intestinal stem cells (ISCs). Accordingly, we performed RNA-seq at the same time point. We calculated a self-organizing map (SOM), integrating data from three replicates of each genotype and condition. Therefore, we analyzed correlated transcription of the gene sets across all samples. Such gene sets form clusters in the SOM (Figure 2A). We identified clusters of genes that are significantly up-(down-) regulated in *Msh2*^−/−^ compared with *Msh2*^+/+^ mice (Figure 2B), while no significant differences were found between radiated and untreated *Msh2*^+/+^ mice and between *Msh2*^+/−^ and *Msh2*^+/+^ mice. Among those genes up-regulated in *Msh2*^−/−^ compared with *Msh2*^+/+^ mice (cluster I) are goblet cell markers such as *Tff3* and *Crip1*, and among those down-regulated (cluster II) are Paneth cell markers such as *Lyz1* and *Defa21* and MHC-II genes such as *H2-Aa, H2-Ab1,* and *H2-EB1*. The latter dominates the gene set enrichment Z- (GSZ) score of the gene ontology (GO) gene set ‘Immune response’ (Figure 2C). Strikingly, we did not find significant changes in the average transcription of G1 genes in irradiated *Msh2*^+/+^ and *Msh2*^−/−^ mice compared with untreated *Msh2*^+/+^ mice. Most of the G1 genes are part of the cluster of invariant transcription (cluster III, Figure 2D). This cluster also contains ISC marker genes such as *Lgr5* and *Ascl2*, genes of the Wnt-pathway, and common reference genes. While transcription of cluster III genes ranges from repressed to strongly expressed, nearly all G1 genes show low transcriptional activity, typical for H3K27me3 target genes (compare Figure S2B).

Our results raise the question of how such a constant transcription can emerge under H3K4me3 recruitment. In the following, we address this question together with those about the origin and function of G1 gene regulation using an *in silico* model approach. 

### 2.2. Results of Computational Modelling

Before we provide results on G1 gene-specific regulation, we introduce general features of our model of epigenetic regulation [10].

#### 2.2.1. General Model Properties I: Analytic Solutions 

Recently, we have introduced a mathematical framework that allows simulating promoter DNA methylation under homeostasis and during DNA repair [10]. Here, we utilize this framework in order to analyze the specific conditions consistent with the experimentally determined histone modification changes following irradiation and loss of *Msh2*. For this purpose, we first identified parameters of a model gene whose promoter shows low DNA methylation and is associated with H3K27me3 modified histones.

The model gene is assumed to be part of a transcription factor (TF) network that confers a stable activation/repression to it, which is described by a regulation factor F_TF_. We calculated equilibrium histone and DNA methylation states solving the self-consistent equations describing the epigenetic machinery (Supplementary II: Equations (S1)–(S3)). Thereby, the histone and DNA methylation levels are quantified by the fraction of modified nucleosomes (*m_4_, m_27_*) and of methylated CpGs (*m_CpG_*) associated with the promoter, respectively. Transcription values *T* associated with the equilibrium methylation states, which are also in accordance with equilibrium transcription (Supplementary II: Equation (S4), $\dot{T}\;$= 0), specify the regulatory states of the gene. A description of the interactions assumed is given in the Supplementary II (Figure S3). 

**Figure 3:**
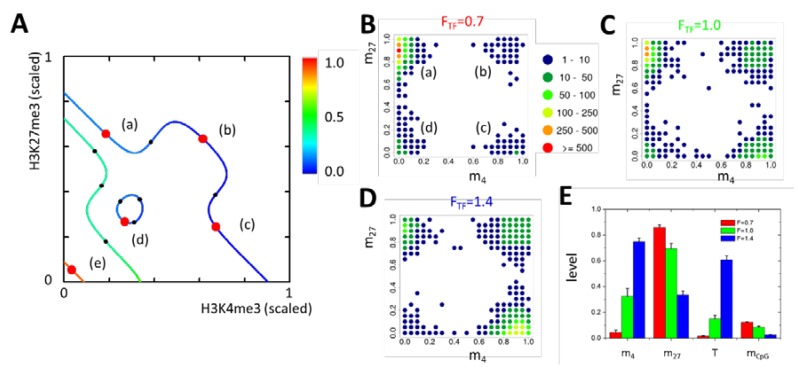
Epigenetic steady-state conditions. (**A**) Single gene states in the H3K4me3–H3K27me3 phase space. The epigenetic machinery defines a set of states that can potentially become occupied (lines, color code: DNA methylation level). The action of the transcriptional machinery is only consistent with a few of them (red and black dots). Beside eight unstable solutions (black dots), five stable states (red dots) are observed: one H3K27me3 (a), one bivalent (b), one H3K4me3 (c), and two unmodified states. Among the latter are one with low (d) and one with high (e) DNA methylation. The modification levels are non-linearly scaled: x_k_ = 0.4 – 0.05 log_10_ ((m_k,max_ – m_k_)/m_k_), k = 4, 27. (**B–D**) Large perturbations enable reversible transitions between locally stable states. Thus, a steady-state distribution is observed (total cell count: 5000). With increasing transcriptional activation F_TF_ of the model gene, H3K4me3 states become more frequent. The high DNA methylation state (e) is not reached, that is, unmodified states are all associated with low DNA methylation. (**E**) H3K4me3 (m_4_), H3K27me3 (m_27_), DNA methylation (m_CpG_), and transcriptional (T) level averaged over a time period of 50τ (average cell cycle time: τ = 1 day, [29]). Averaging was started after an equilibration time of 50τ.

In a broad parameter range, the model has multiple solutions, that is, different regulatory states exist in parallel. Figure 3A shows an example where five regulatory states (red circles) are stable at the same time. The states comprise an H3K27me3 state (a); a bivalent H3K27me3/H3K4me3 state (b); an H3K4me3 state (c); and two unmodified states, one with low DNA methylation (d) and one with high DNA methylation (e). They are part of the states that are consistent with the properties of the epigenetic machinery (lines). The parameter set {A} applied is given in the Supplementary II (Table S2).

#### 2.2.2. General Model Properties II: Steady State Epigenetics

The stability of the epigenetic states is a local one, that is, ongoing perturbations are capable of inducing state changes. Thus, in a dynamic simulation, a population of cells adopts a quasi-steady state distribution. This behavior is in agreement with experimental findings in mouse embryonic stem cells [24]. ‘Dilution’ of histone modifications following each cell division represents an example of a particular strong perturbation. The modified histones of the mother cell are randomly distributed onto the daughter cells and are supplemented by newly synthesized ones [25]. Cooperativity of histone methyltransferase (HMT)-binding then enables the re-establishment of the modification state of the mother cell [26], as long as the perturbed modification states did not leave the attractor of it. Otherwise, modification is lost and the state changes. Such changes are reversible owing to ongoing stochastic histone (de-) modification events. Starting simulations of model gene dynamics in the attractors of states (a–d), that is, with low DNA methylation (m_CpG_<m_CpG,C_), a population of cells adopts a quasi-steady state distribution largely excluding state (e) (Figure 3B–D). Mean values m_4_, m_27_, m_CpG_, and T, averaged over all cells, are given in Figure 3E and Supplementary III (Figure S4A). Depending on the regulatory impact of the TF network, which is given by the regulation factor F_TF_, most cells occupy an H3K27me3 (F_TF_ = 0.7), a low expressing bivalent (F_TF_ = 1, T < 0.2) or a high expressing bivalent state (F_TF_ = 1.4, T > 0.6). On a long time-scale, the distribution underlies a drift, as more and more genes enter the highly methylated state (e) [27,28].

This drift requires that the attractor of state (e) is reached (m_CpG_ > m_CpG,C,_ Figure 3A). In our simulations, we suppress such behavior by neglecting fluctuations of the methylation state of individual CpGs and perform a deterministic update (Supplementary II, Equation (S3)). Accordingly, for the parameter set applied, un-methylated (a–d) and methylated states (e) become separated. Thus, decreasing m_CpG_ of the initial state to m_CpG_ > m_CpG,C_, the model gene approaches state (e), where it remains (Supplementary Figure S4B). On the basis of these results, we assume that, under reference conditions, H3K27me3 target genes with low promoter methylation can be described by the parameter set {A} with F_TF_ ≤ 1 and m_CpG_ < m_CpG,C_. Thereby, we consider the H3K4me3 level observed under these conditions (m_4_<0.5) to be too low for reliable experimental detection.

#### 2.2.3. Definition of G1 Model Genes via DNA–HMT Interaction 

**Figure 4:**
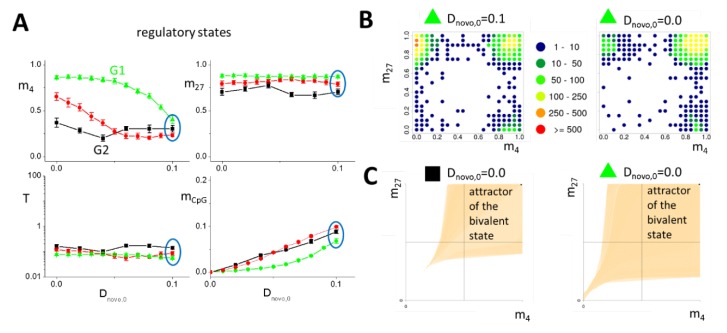
Regulatory states at decreased *de novo* DNA methylation activity. (**A**) Regulatory states depend on CpG binding of HMTs. At D_novo,0_ = 0.1, G1 (ε_BS_ = 5.5, green) and G2 genes (ε_BS_ = 5.0, black) occupy similar regulatory states. A decrease of D_novo,0_ at ε_BS_ = 5.5 leads to an immediate recruitment of H3K4me3, while at ε_BS_ = 5.2 (red), such an increase is observed below D_novo,0_ = 0.05 only. It nearly vanishes at ε_BS_ = 5.0 (black). Error bars: SD owing to state fluctuations over time. (**B**) Histograms of occupation frequency of the (H3K4me3, H3K27me3) states for ε_BS_ = 5.5. At D_novo,0_ = 0.1, a broad state distribution is seen (left), while at D_novo,0_ = 0.0, the population becomes homogeneous and G1 model genes are bivalent, that is, H3K4me3 and H3K27me3 modified, in most of the cells (right). (**C**) Changes of the occupation frequency with ε_BS_ (5.0 (left), 5.5 (right), D_novo,0_ = 0.0) originate in a changed size of the attractor of the bivalent state (beige area).

Both G1 and G2 genes are H3K27me3 targets under reference conditions, but only G1 genes recruit H3K4me3 following genomic stress. Thus, regulatory differences must exist that distinguish the genes of both sets. G1 genes show a higher CpG, GpC fraction compared with G2 genes (Figure 1C), explaining their lower promoter DNA methylation [20]. To account for this difference, we considered a higher interaction energy ε_BS_ between CpG-binding HMTs and the un-methylated promoter for G1 (ε_BS_ = 5.5) compared with G2 genes (ε_BS_ = 5.0, setting in previous simulations). Despite these differences, for otherwise identical parameters, we found the average regulatory states (m_4_, m_27_, T, m_CpG_) of the two types of genes (Figure 4A) as well as the underlying state distributions (Figure 3C and Figure 4B) to be very similar. In particular, G1 genes still resample typical properties of H3K27me3 modified genes.

#### 2.2.4. Reduced DNA Methylation Activity Enables H3K4me3 Recruitment to Promoters of G1 Model Genes

Our model describes H3K4me3 recruitment as an autocatalytic process that becomes amplified for increasing transcription, improving the accessibility of the chromatin, and/or reducing the DNA methylation level (Supplementary II, Equation (S1)). H3K4me3 recruitment by G1 genes owing to increased transcription contradicts our RNA-seq results. Moreover, while increasing H3K4me3, it would in parallel decrease H3K27me3, inducing an H3K4me3 state, in contrast to our former ChIP-seq results [20] (compare Supplementary III, Figure S4A). 

In contrast, reduced DNA methylation stabilizes both H3K4me3 and H3K27me3, consistent with the bivalent state of G1 genes observed following genomic stress. Thus, we assumed reduced promoter DNA methylation in our simulations and tested whether this assumption is consistent with a different response of G1 and G2 genes. There are several options to lower DNA methylation, including changes in the activity of the maintenance and *de novo* DNMTs, or the TET-pathway. In our model, changing the maintenance DNMT activity has only marginal effects on the DNA methylation status of weakly methylated genes (Supplementary IV, Figure S5A). Moreover, G1 genes appear to be not actively de-methylated by the TET-pathway (Supplementary IV, Figure S5B). Thus, we assumed that *de novo* DNMTs trigger H3K4me3 recruitment by G1 genes.

Simulating a reduced *de novo* DNA methylation by decreasing the maximum *de novo* methylation rate, D_novo,0_, the response of G1 and G2 genes indeed was different. For values of ε_BS_ = 5.5 (G1), the H3K4me3 level immediately increases, decreasing D_novo,0_ from its reference value 0.1 to 0.0 (Figure 4A). However, for ε_BS_ = 5.2, an increase of the H3K4me3 level is observed below D_novo,0_ < 0.05 only and, for ε_BS_ = 5.0, the modification level remains constant. In all settings, a high H3K27me3 level was observed. Thus, according to our simulation results, low *de novo* methylation activity following genomic stress is a potential explanation of G1 gene-specific recruitment of H3K4me3. Notably, a similar G1-specific recruitment can be achieved by improving chromatin accessibility. Also, in this scenario, H3K4me3 recruitment is associated with a decreased DNA methylation (Supplementary V, Figure S6). Both explanations cannot be validated directly by DNA methylation or ATAC sequencing, respectively, as reduced DNA methylation and open chromatin are generally expected following H3K4me3 recruitment (feedback, see Supplementary II Figure S3). So, we looked for more specific effects of such changes.

#### 2.2.5. Conserved Transcription of G1 Model Genes under H3K4me3 Recruitment

Further support for our hypothesis on H3K4me3 recruitment comes from the transcriptional regulation seen under the proposed scenario (Figure 4A). Decreasing D_novo,0_, the transcription of the G1 model gene remains largely stable for ε_BS_ = 5.5 despite H3K4me3 recruitment, in agreement with our experimental findings. Thereby, the bivalent histone state becomes more attractive, as indicated by the histograms for the occupation frequency of the regulatory states (Figure 4B). Differences in the occupation frequency originate in an extension of the attractor of the bivalent state (Figure 4C). Therefore, the following consequences are posed: In many cells, G1 genes switch from the H3K27me3 state (a) into the bivalent state (b). This increases their expression. However, in a few cells, G1 genes reach the bivalent state (b) also from the H3K4me3 state (c). This decreases the G1 gene expression within these cells. The decrease from an H3K4me3 to a bivalent state (ΔT > 1) is much stronger than the increase from an H3K27me3 to a bivalent state (ΔT < 0.1). As a consequence, the transcription, averaged over all cells, can be approximately balanced. Thus, our model predicts, in addition to changes in the *de novo* DNA methylation activity, an increased homogeneity of the cell population regarding the transcription of G1 genes following irradiation or loss of *Msh2*. This homogeneity can be verified by single cell RNA-seq. The predicted induction of actual bivalence of G1 genes can be verified as well, for example, by applying sophisticated histone ChIP-seq [30].

#### 2.2.6. Following Genomic Stress, G1 Model Genes Are Protected Against Promoter DNA Hyper-methylation

**Figure 5:**
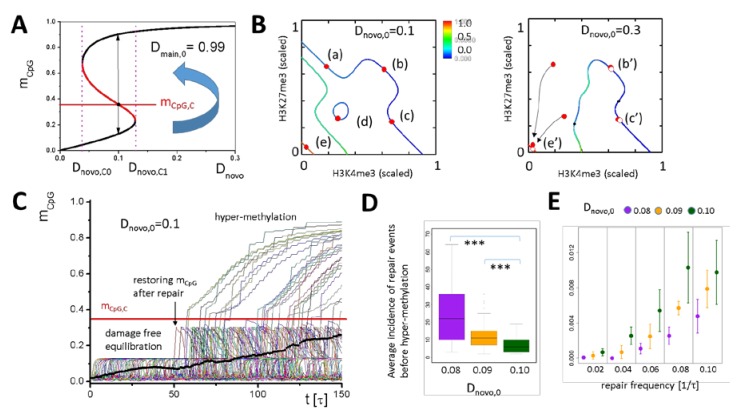
Hyper-methylation induced by increased *de novo* DNA methylation activity. (**A**) The DNA methylation level, m_CpG_, depends on the *de novo* DNA methylation activity. Within the bistable region [D_novo,C0_ D_novo,C1_], the threshold level m_CpG,C_ defines whether m_CpG_ reaches the high or low methylation state. Increasing the *de novo* DNMT activity above D_novo,C1_ renders low DNA methylation states unstable. (**B**) At D_novo,0_ = 0.3, this applies to the H3K27me3 and the unmodified state with low DNA methylation (stable states at 0.1: solid circles, at 0.3: open circles, color code lines: DNA methylation level). (**C**) Simulation of ongoing DNA repair (onset at t = 50τ, frequency: 0.04/τ). A temporary increase of D_novo,0_ to 0.3 in individual cells (thin lines) increases m_CpG_. In most cells, the original methylation level is restored after repair in a few cell cycles. In the case of repeated repair, m_CpG_ can exceed m_CpG,C_, leading to DNA hyper-methylation. Thick line: average DNA methylation. (**D**) The average incidence of repair events before hyper-methylation is higher at lower initial D_novo,0_ (*** *p*-values < 1e-5, Kolmogorov–Smirnov test). (**E**) Accordingly, the hyper-methylation probability is lower at a lower D_novo,0_, independent of the repair frequency (errors: SD).

During DNA repair, reduced lysine-specific demethylase 2B (KDM2B) locally increases the *de novo* DNMT activity [12], increasing the probability of DNA hyper-methylation [31]. As stable H3K4me3 modified promoters are protected from DNA methylation [13], we hypothesized that H3K4me3 recruitment by G1 genes can significantly improve their resistance against hyper-methylation during DNA repair. We model increased *de novo* DNMT activity during DNA repair, increasing the parameter D_novo,0_ (Supplementary II, Equation (S3b)) [10]. This increase affects the solutions for stable epigenetic states. Above the threshold value D_novo,C1_ (Figure 5A), the solutions of Equations (1)–(4) change qualitatively. Figure 5B compares the solutions of the system for D_novo,0_ = 0.1 and 0.3 (F_TF_ = 1). At D_novo,0_ = 0.3, the states (b), (c), and (e) slightly move in the H3K4me3–H3K27me3 state-space towards (b’), (c’), and (e’), but remain locally stable, while the H3K27me3 (a) and the intermediate state (d) vanish. For D_novo,0_ larger than the threshold D_novo,C1_, the gene definitely enters the unmodified, highly DNA methylated state (e’) and remains in this state. The equilibrium is approached exponentially (Supplementary Figure S5C). In simulations of DNA repair, we considered an increase of D_novo,0_ for a defined time period Δt ≤ τ only. 

DNA damage is assumed to randomly hit a model gene with a constant probability per cell division and cell defining the repair frequency. In any case, the damage is assumed to be fully repaired during the cell cycle in which it occurs [10]. We compared the response of a G1 model gene with reduced (D_novo,0_ < 0.1, H3K4me3 recruiting) and reference *de novo* DNA methylation activity (D_novo,0_ = 0.1). For this purpose, we simulated 100 cells, starting from stochastically acquired states of the G1 model gene, and recorded the state of the gene in all cells over time. Details can be found in Supplementary VI. If the gene is subject to repair, it increases its DNA methylation level, however, in most cases, it restores it after repair within a few cell cycles (Figure 5C), in agreement with experimental findings [32]. Only a few genes start hyper-methylation. We found that G1 model genes, where D_novo,0_ was reduced by 20%, hyper-methylate with a significantly lower probability compared with reference G1 genes (Figure 5E/F). Thus, following genomic stress, G1 model genes are protected from becoming hyper-methylated during a repeated, temporary increase of the *de novo* methylation activity. This suggests that epigenetic changes observed for G1 genes following DNA damage function to counteract part of the regulatory changes during DNA repair. Notably, below a threshold *de novo* DNA methylation activity (D_novo,C0_), a high methylation state no longer exists and, accordingly, hyper-methylation cannot occur at all.

#### 2.2.7. Behind G1 Model Genes: Induced H3K4me3-H3K27me3 Bivalency

**Figure 6:**
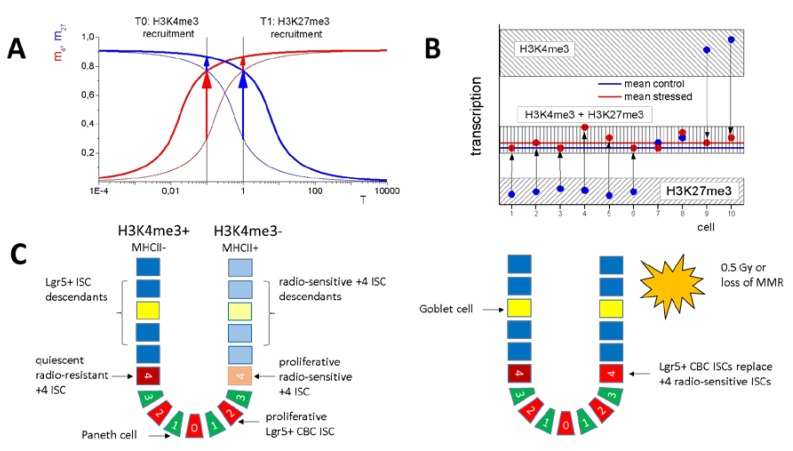
Hypothetic regulation scenario following DNA damage. (**A**) Lower promoter DNA methylation improves both H3K4me3 and H3K27me3 recruitment (thick curves) compared with the reference system (thin curves). Accordingly, low expressing (T0) G1 genes with low H3K4me3 and high H3K27me3 strongly recruit H3K4me3 (long red arrow), while higher expressing (T1) genes with high H3K4me3 and low H3K27me3 under the same conditions strongly recruit H3K27me3 (long blue arrow). (**B**) Thereby, T0 refers to the average expression of G1 genes. Actually, G1 genes occupy different modification states in *Msh2^+/+^* mice, and reaching the H3K4me3–H3K27me3 bivalent state comprises transcriptional up- and down-regulation, explaining the on average stable transcription. (**C**) Intestinal stem cell (ISC) regulation following DNA damage in intestinal crypts: replacement of radio-sensitive +4 ISCs (MHCII+) by Lgr5+ crypt base columnar (CBC) ISCs (MHCII–), after a single irradiation with 0.5 Gy or loss of *Msh2*, changes the average epigenetic states of the descendants. We assume that G1 gene promoters are devoid of H3K4me3 in +4 radio-sensitive ISCs (H3K4me3-state), while they carry this modification in Lgr5+ CBC ISCs (H3K4me3+ state). Radio-resistant +4 ISCs survive and remain quiescent. Numbers indicate cell position in the crypt (0 = crypt base).

So far, we focused on H3K27me3 target genes recruiting H3K4me3 following irradiation and/or *Msh2* loss. However, there is also a large group of H3K4me3 target genes that recruit H3K27me3 [20]. According to our model assumptions, these genes represent transcriptionally activated genes under the same regulation as described here (F_TF_ > 1, see Figure 3B–E). In order to illustrate this hypothesis, Figure 6A shows a sketch of the kind of regulation assumed (average regulatory states). In the model, promoter demethylation enforces both H3K4me3 and H3K27me3 recruitment, that is, stabilizes bivalent genes. As a consequence, it induces H3K4me3 recruitment to low expressing, H3K27me3 target genes (as described), and recruitment of H3K27me3 to high expressing, H3K4me3 target genes. Thus, our model explains the seemingly contradictory behavior of long-term gene regulation following DNA damage.

## 3. Discussion 

Long-term changes in the epigenome induced by genomic stress can be associated with an increased risk of tissue transformation [6]. Recently, we studied such changes following irradiation and loss of *Msh2* [20]. We frequently observed recruitment of H3K4me3 to histones associated with H3K27me3 target genes. Here, we show that H3K9me3 is not involved in this regulation. Using an *in silico* model approach, we suggest that the changes originate in a reduced promoter DNA methylation following genomic stress. This assumption consistently explains our observation that H3K4me3 recruitment occurs without changes in the transcription level of the associated genes (Figure 6B).

TP53 is a master regulator of DNA damage response [33]. Thus, the assumed DNA de-methylation following irradiation and loss of *Msh2* might be induced by *Tp53* activation [34]. Actually, *Tp53* activation by irradiation is considered to suppress both maintenance and *de novo* DNMT activity [35]. Accordingly, it might induce G1 gene promoter DNA hypo-methylation and H3K4me3 modification. In addition, TP53 has been shown to strengthen TET1 activity in embryonic stem cells [34]. Thus, related hydroxy-methylation might contribute to G1 gene promoter hypo-methylation and H3K4me3 modification in accordance with its function in *de novo* formation of bivalency at CpG islands [36].

Both hypo-methylation scenarios are consistent with experimental findings of a global DNA hypo-methylation following irradiation in the murine thymus with 0.5 Gy, which was accompanied by reduced DNMT1 and DNMT3a expression [37]. However, this study reports DNA methylation changes at short time scales of hours only. Thus, the question remains of how such regulation, being active during DNA damage repair, confers long-term changes. A potential scenario might be frequent hyper-methylation of the DNMT3a promoter or DNMT3a mutation following irradiation and associated DNMT3a down-regulation. In the intestine, affected cells then have to survive monoclonal conversion, that is, have to win clonal competition [38,39]. Nevertheless, high TP53 confers slow cell cycle re-entrance after DNA repair [40]. Thus, clones with high TP53 will rarely win the competition. Accordingly, we favor a different explanation of the long-term epigenetic effects.

The small intestine contains different functional stem cell populations [41,42]: the crypt base columnar (CBC) cells, actively cycling cells that strongly express Lgr5, and the so-called +4 cells that are situated above the crypt base around cell position 4. The Lgr5+ CBC population is radio-resistant [43]. The +4 ISC population divides into a radio-sensitive, proliferative; and a radio-resistant, quiescent subpopulation. The radio-sensitive population is also sensitive to insults such as tamoxifen treatment [44] and can thus be considered as stress-sensitive in general. The radio-resistant population survives such treatment and can be considered as stress-resistant.

Already, low radiation (<0.5 Gy) eliminates the entire radio-sensitive +4 ISC population [45]. Such perturbations, however, appear to be insufficient to activate *Bmi1*-expressing, quiescent reserve ISCs [44]. Thus, 0.5 Gy radiation leads to a replacement of radio-sensitive +4 ISCs by radio-resistant CBC ISCs (Figure 6C). Considering that +4 radio-sensitive ISCs substantially contribute to lineage tracing events under homeostasis [44], long-term changes in H3K4me3 recruitment, as observed in our former experiments [20], can be explained by assuming that the progeny of the CBC ISCs show lower *de novo* DNMT activity compared with the replaced +4 ISCs. Notably, the presence of stem cell subpopulations with different DNA methylation profiles has been demonstrated recently for hematopoietic stem cells [46]. The radio-resistance of the CBC ISCs might originate, at least in part, in the increased H3K36me3 modification seen in a large number of genes [20]. The cause of these modification changes remains, however, speculative.

Support for the assumption of a similar ISC replacement following *Msh2* loss comes from single-cell RNA-seq data identifying three ISC subpopulations [47]. Two of them have been shown to strongly express MHC-II genes, while the third ISC subpopulation and all other epithelial cells in the small intestine of mice do not. In our data, we found the expression of a subgroup of MHC-II genes to be among those most strongly down-regulated in *Msh2^−/−^* mice. This suggests that MHCII+ ISCs become replaced by MHCII- ISCs in these mice. In addition, we found that the *Tff3* gene, known to be associated with radiation protection [48], becomes upregulated following *Msh2* loss, consistent with the expansion of a stress-resistant ISC population.

Four weeks after irradiation, changes of MHC-II gene expression are only weak (Figure 2C). Actually, following a single radiation hit, the +4 ISC population is known to regenerate from CBC ISCs [43]. This process can require weeks [49]. In order to explain the observed changes in H3K4me3 profiles following irradiation, we assume that the typical expression of MHCII+ ISCs is re-established within four weeks, while epigenetic states of these cells require longer times to regenerate.

In our simulations, a similar effect on H3K4me3 and H3K27me3 modification as conferred by low *de novo* DNA methylation activity is observed, assuming high chromatin accessibility. Thus, G1 gene properties can also be explained by higher chromatin accessibility in CBC compared with +4 ISCs. Reports on heterochromatic regions alone indicate a relaxation after irradiation exposure and re-condensation during repair, whereas euchromatin seemed to be unaffected or behave contrarily [50]. Also regarding these changes, TP53 is an important player, being capable of increasing chromatin accessibility in close vicinity to enhancers [51]. These observations are in agreement with improved H3K4me3 recruitment to H3K27me3 target genes that are frequently associated with facultative heterochromatin. However, TP53-associated effects on DNA methylation were detected on a time-scale of a few days and not of weeks.

## 4. Conclusions

Our *in silico* simulations suggest that stress-resistant ISC subpopulations are protected from promoter DNA hyper-methylation in the course of DNA repair, preventing accelerated epigenetic aging of the entire tissue during periods of prolonged stress. This potential assigns the epigenetic profiles, acquired by these cells, a clear function. To our best knowledge, long-term changes following moderate DNA damage in the intestine have been not studied so far. In general, knowledge about the time scales required to re-establish stem cell homeostasis after damage and for the regeneration of reserve stem cell pools is rare. *In silico* models can support related investigations, providing substantiated hypotheses about epigenetic regulation of stem cells in regenerative tissue.

## 5. Material and Methods

The experimental design has been described in our preceding study [20] and is attached as Supplementary Material I. A description of the model of epigenetic regulation of transcription [10] is provided in the Supplementary Material II. 

## 6. Data Availability

The mouse jejunum data presented in this work can be obtained in the Gene Expression Omnibus (GEO) repository (https://www.ncbi.nlm.nih.gov/geo) under the super series accession number GSE146520. This series comprises the mouse jejunum RNA-seq data (individual accession number GSE146518) and the mouse jejunum ChIP-seq data (individual accession numbers GSE146519 and GSE128842 (Herberg et al. [20])).

Further sequencing data supporting the conclusions of this article were obtained from the GEO repository and the ArrayExpress Archive of Functional Genomics Data (https://www.ebi.ac.uk/arrayexpress). The data by Mikkelsen et al. [23] are deposited in GEO under the series accession number GSE8024. The data by Kim et al. [52] are stored in ArrayExpress under the accession number E-MTAB-5202. 

## Supplementary Materials

The following are available online at https://www.mdpi.com/1422-0067/21/6/1941/s1.

## Abbreviations

ISC	intestinal stem cell
H3KXme3	histone 3 lysine X tri-methylation
DNMT	DNA methyltransferase
HMT	histone methyltransferase
SOM	self-organizing map
CBC	crypt base columnar
HCG, ICG, LCG promoter	promoter with high, intermediate, low CpG density
TF	transcription factor
