# Supplementary material for: Fighting Against Promoter DNA Hyper-Methylation: Protective Histone Modification Profiles of Stress-Resistant Intestinal Stem Cells
Source: Int J Mol Sci. 2020 Mar 12;21(6):1941. doi: 10.3390/ijms21061941 (PMC7139626; doi:10.3390/ijms21061941)
Supplement: Supplementary file 1 [file ijms-21-01941-s001.pdf]

Supplementary Material

I. Materials and Methods (Herberg et al. [1])

Collection of tissue samples: The mouse strains B6.SJL-Tg(Villin-Cre)997Gum/J and B6.Cg-Msh2tm2.1Rak/J mice were obtained from The Jackson Laboratory (Bar Harbor, USA). By crossing both, the conditional Msh2 allele has been placed under the control of the Villin-Cre transgene. Mice were genotyped as described [2]. $VC^{+/?}Msh2^{LoxP/LoxP}$ ($Msh2^{-/-}$), $VC^{+/?}Msh2^{LoxP/+}$ ($Msh2^{+/-}$), and $VC^{-/-}Msh2^{LoxP/LoxP}$ ($Msh2^{+/+}$) mice were bred under specific pathogen-free conditions. The proximal jejunum, defined as the first third of the intestine after the ligament of Treitz, was dissected from each mouse (3 per genotype and condition) and shock frozen without delay. Tissue segments were stored in liquid nitrogen until further use.

Radiation of mice. We obtained ethics approval from the Landesdirektion Leipzig (TVV 53/14) for radiation of mice. Three month old mice were placed in the radiation unit (X-ray generator, Gulmay D3225, round tube 170 mm, radiation level at 2.5 cm: 1.068 Gy/min) and irradiated with a total dose of 0.5 Gy. All mice were killed 28 days after radiation.

ChIP-seq, data pre-processing and analysis. Chromatin was prepared from up to 50 mg jejunum using SDS Shearing Buffer and the truChlP Tissue Chromatin Shearing Kit (Covaris, Brighton, UK). Chromatin immunoprecipitations (ChIPs) were run on the IP-Star compact system using the Auto iDeal ChIP-seq kit for histones (Diagenode, Seraing, Belgium). Library preparations were performed according to the TrueSeq LT PCR free or TruSeq Nano DNA Kit instructions (Illumina, San Diego, USA). Sequencing was performed on the Illumina HiSeq 4000 with 76 bp paired-end sequencings.

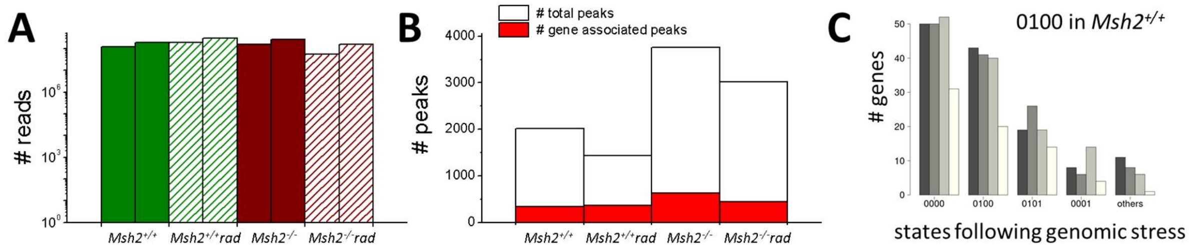

Fig. S1: Additional results: H3K9me3 ChIP-seq. A) Number of high quality reads detected in the different mice. B) Numbers of consensus peaks called and of those peaks being associated with a gene. C) Modification changes of genes exclusively H3K9me3 modified in $Msh2^{+/+}$ mice (0100). These genes do not recruit H3K4me3. Most frequently these genes become de-modified, reaching the histone H3 modification state (0000).

The sequenced reads underwent a quality trimming using cutadapt [3] to ensure a minimum read length of 40 bases and a Phred quality score above 20. Quality reports were generated and analyzed using Fastqc [4]. The high-quality reads (numbers see Fig. S1A) were mapped to the mouse reference genome NCBI37/mm9 using the software tool segemehl 0.2.0 [5]. Subsequently, peaks were identified by applying MACS 1.4.2 [6], with the H3pan data provided by [1] as controls and the following parameter settings: `--nomodel -w --space=30 --bw 300 --pvalue 1e-5 --shiftsize 200`. The peaks were checked for artificial amplification and then used to generate summarized peak lists for each

genotype and treatment. Each list contains the peaks that are either consistently detected in both replicates or have a high reliability (MACS fold enrichment > 5.0) Very few of them are located in blacklisted regions ([7], ≤ 2%). We marked these peaks but did not remove them from the lists. Subsequently, we analyzed whether peaks being located within the promoter region (defined as transcriptional start site (TSS) +/- 1000 bases) or gene bodies (defined as region between the TSS and the last base of the gene). The gene reference list containing 31592 RefSeq genes was taken from UCSC Table Browser. To avoid gender-specific artifacts, we excluded genes and peaks of the X- and Y-chromosome from analysis. If a peak had a minimum overlap of 5% with a promoter region and/or a gene body, it was considered as gene associated and the respective gene to carry the modification (present: 1, absent: 0). Total numbers of peaks and numbers of gene associated peaks are shown in Fig. S1B. Effects of genomic stress on H3K9me3 target genes are quantified in Fig. S1C.

Promoter CpG, GpC density. A core promoter was defined as the 1kb broad region [TSS-500bp, TSS+500bp] consistent with our 5hmC analysis (Supplementary IV). CpG and GpC pairs were quantified counting the fraction of C and G bases that is downstream followed by a G or C base, respectively.

RNA-seq, data pre-processing and analysis. Whole tissue from jejunum was homogenized with the Tissue Lyser (20s at 15 Hz). RNA was prepared following manufacturer's recommendations (Qiagen QIA AllPrep kit) including a DNase I digest and sequenced on an Illumina HiSeq 4000 sequencer with 75bp paired-end, strand-specific, random priming. Raw counts were normalized to the total number of reads resulting in one reads per million (RPM) value per gene. These RPM values were normalized to the genes length (RPKM-reads per kilobase million). Boxplots of RPKM values of selected gene sets are given in Fig.

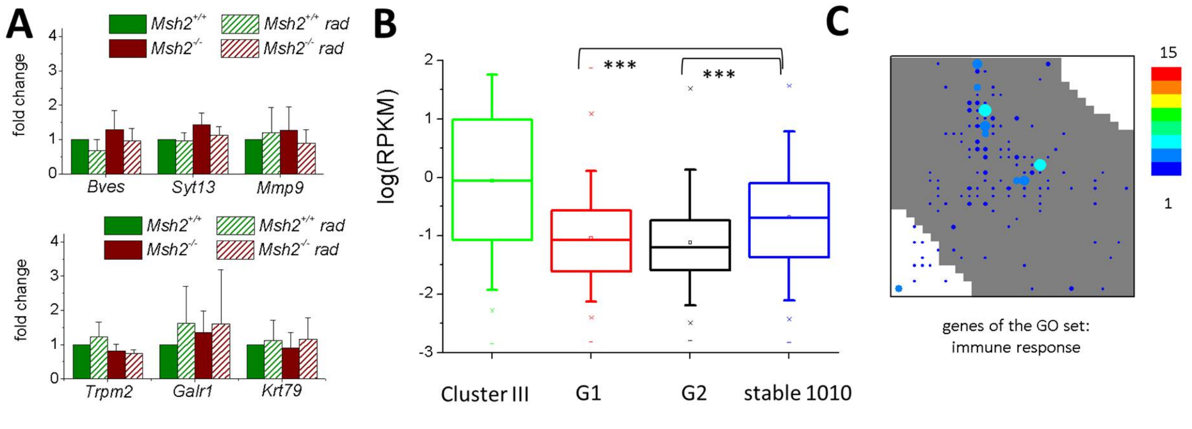

S2B.

Fig. S2: Additional results: gene transcription. A) Quantitative RT-PCR results for genes of cluster III (Fig. 2A). Consistent with RNA-seq results, an invariant transcription is observed for genes of the set G1 (upper box) and G2 (lower box). The fold change in the transcription of all mice (3 replicates of each, error: SD) is shown. B) Boxplot of the transcription values of selected gene sets as determined by RNA-seq in *Msh2*$^{+/+}$ mice. Genes of cluster III show a broad distribution, while the gene sets G1 and G2, being subsets of them, show a low transcription level significantly below that of H3K4me3-H3K27me3 bistable genes. The set sizes are reduced due to incomplete data mapping and selection RPKM>0. *** p<1e-6 (Kolmogorov-Smirnov-test). C) Location of the genes of the GO set 'immune response' in the SOM introduced in Fig. 2 (colors: # of genes per metagene).

The transcription profiles over all samples are then clustered into metagene profiles using SOM machine learning, with each metagene serving as a representative of a cluster of combined profiles. We used a grid of 30 x 30 metagenes and applied default parametrization as implemented in oposSOM [8]. Genes with similar transcription in all samples are assigned to the same or neighboring metagenes; the latter forming clusters. Predefined gene sets are often distributed across different clusters as e.g. the GO set 'immune response' (Fig. S2C).

Quantitative RT-PCR. Intestinal tissue was homogenized in 1ml TRIzol® Reagent (Invitrogen, Germany) and total RNA was isolated according to the manufacturer's instructions. For cDNA synthesis, 1 µg RNA was transcribed using the SuperScript™ IV First-Strand Synthesis System (Invitrogen). Relative levels of mRNA were quantified using an Applied Biosystems 7500 Real-Time PCR System with the ΔΔCt method. Expression of specific genes was normalized to murine *Rps29* and fold changes of biological replicates were averaged. All reactions were performed by using technical replicates. Primer sequences are provided in Table S1. Results are summarized in Fig. S2A.

Table S1: Primer sequences

Gene name	Primer pair sequence (5'-3; 'for/rev)
Gfra3	GGACCCTTTGTGCAGATCAC<br>GGTGCAGCTTAAGGCAACAG
CalcB	CCAGGAAGAAGGTTACATAAAGTTG<br>GCTGGATGGCTCTTGGAGAA
Resp18	CGACATAAACGCCCACGATG<br>AGAACATGCCTTCGGGTACAA
Bves	GCGAAAAATTCCAGGTCACCAT<br>ACAGAAAAGGCTCTGACTCCAG
Syt13	GGGCAAGGATGTGTCTGTCA<br>TCCACACGGGGTTGATCTTG
Mmp9	CAGCCGACTTTTGTGGTCTTCc<br>GCGGTACAAGTATGCCTCTGC

II. Basic assumptions and equations of the epigenetic regulation model (Thalheim et al. [9])

Fig. S3: Regulatory network assumed in the model [9].

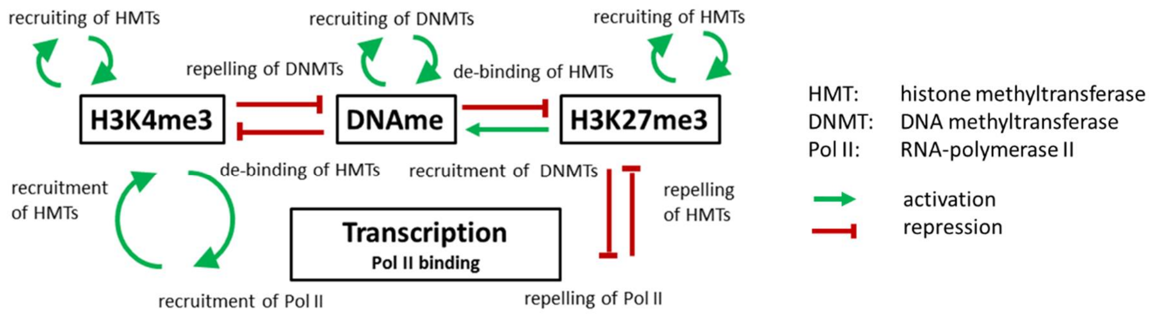

Regulation of histone modification: The binding probabilities of the HMTs, Θ^K (K=4, 27), are calculated assuming a positive feedback between the presence of the histone mark K and

the recruitment of its HMTs. Such a feedback has been demonstrated for both H3K4me3 and H3K27me3 [10]. According to this assumption, the binding probability of the HMT of histone mark K depends on the fraction m_K (K=4, 27) of modified nucleosomes of the N_H cooperative nucleosomes associated with the promoter and is given by:

$$\Theta^K = \frac{1}{1+\exp(\varepsilon_0^K + m_{DNA}\cdot\varepsilon_{BS}^K + m_K\cdot\varepsilon_{HM}^K\cdot N_H + \lambda_K\cdot\log(T))}$$
(S1)

Here, ε_0^K is the ground enthalpy per bound HMT complex and ε_{BS}^K and ε_{HM}^K are the free energies of HMT binding to DNA and to histone mark K, respectively. They are specific for histone mark K and are scaled by the Boltzmann unit. The factor λ_K is (-1) for H3K4me3 and (+1) for H3K27me3. Assuming a dynamic equilibrium between modification and de-modification, Θ^K is also given by:

$$\Theta^K = \frac{C^K\cdot m_K}{1-m_K}$$
(S2)

where C^K is the de-modification constant $C^K = k_{de}/k_{mod}$; i.e. the ratio between the de-modification rate k_{de} and the modification rate k_{mod} for the respective modification.

Analytical solutions of the histone methylation machinery (m_4, m_{27}) consistently solve Equ. S1 and S2. In dynamic simulations, individual histones are either modified with the probability $k_{mod}\cdot\Theta^K dt$ or de-modified with probability $k_{de}dt$ per time step dt.

The changes Δm_{CpG} of the DNA methylation subsequent to cell division (to reach a balance between maintenance and *de novo* DNA methylation) are than described by:

$$\Delta m_{CpG} = D_{novo}(m_4, m_{27})(1 - m_{CpG}) - \left(1 - D_{main}(m_{CpG})\right) m_{CpG}.$$
(S3)

We assume that the DNMT1 binding probability to the promoter depends on the methylation level m_{CpG} of the CpGs at this promoter:

$$D_{main} = D_{main,0}/\left(1 + \exp\left(E_{m,0} + E_{m,1} m_{CpG}\right)\right).$$
(S3a)

This binding is controlled by two energy constants $E_{m,0}$ and $E_{m,1}$ describing accessibility for and binding of DNMT1 to methylated CpGs of the promoter, respectively. According to this feedback, the DNA methylation becomes bistable for defined parameter sets. The range of bistability is controlled by: i) the energy constants ($E_{m,0}$, $E_{m,1}$), ii) the maximum probability of maintaining a methyl-CpG in the daughter ($D_{main,0}$), and iii) the effective *de novo* DNA methylation probability $D_{novo}(m_4, m_{27})$. The latter depends on the histone modification states of the gene under consideration [11]:

$$D_{novo} = D_{novo,0}\left(1 + \exp\left(-E_{CpG}^{27}\cdot m_{27}\right)\right)/\left(1 + \exp\left(E_{CpG}^{4}\cdot m_4 - E_{CpG}^{27}\cdot m_{27}\right)\right).$$
(S3b)

where E_{CpG}^4 and E_{CpG}^{27} are energies modulating the binding of *de novo* DNMTs to the promoter in presence of the respective modification.

The methylation states of the regulatory states of the systems (m_4, m_{27}, m_{CpG}) are given by $\Delta m_{CpG}=0$.

Regulation of gene expression: We assumed gene transcription to depend on the histone modification levels m_4 and m_{27} of the gene promoter. Accordingly, the transcription of the individual genes is calculated by solving:

$$\frac{dT}{dt} = \frac{P_{max} \cdot (m_0 + m_4) \cdot (1 - m_{27})}{1 + \frac{P_{max} - 1}{F_{TF} \cdot F_{auto}}} - \delta T. \quad \text{(S4)}$$

Here, P_{max} is the maximum promoter activity and δ the transcript degradation rate. F_{TF} and F_{auto} are the regulation factors for the TF-network and for the auto-regulation of the gene, respectively. Details about the underlying regulatory principles of the TF-network, which are based on thermodynamics, can be found in [12]. $F_{auto}=1$ is set throughout the study. The constant m_0 <<1 ensures that genes can be transcribed with a small rate also if the promoter is devoid of H3K4me3 ($m_4=0$).

Analytical solutions for the transcriptional machinery T* require $(dT/dt)_{T^*}=0$. Thus, epigenetic states that are consistent with the transcription machinery represent a small subset of the solutions of Equations S1-3. In dynamic simulations, in addition to the fluctuations of the histone modification states described above, cells underwent a dilution of histone modification during cell division. Modified nucleosomes of the mother cell are randomly distributed onto the daughters and complemented with unmodified ones. This dilution is the main reason for instable modification states, i.e. spontaneous de-modification [13].

Table S2: Parameter set A (X,Y,Z)

**Parameter**	**Value**	**Description**
$\varepsilon_0^4, \varepsilon_0^{27}$	9.0, 11.0	ground enthalpy per bound HMT*
$\varepsilon_{BS}^4, \varepsilon_{BS}^{27}$	G1: -5.5,- 5.5<br>G2: -5.0, -5.0	free energy of CpG binding*
$\varepsilon_{HM}^4, \varepsilon_{HM}^{27}$	-0.4, -0.4	free energy of histone binding*
C^4, C^{27}	0.1, 0.1	de-modification constant
N_H	20	number of cooperative nucleosomes

Parameter set {X} of the histone modification machinery. *scaled by the Boltzmann unit.

**Parameter**	**Value**	**Description**
P_0	100	Maximum transcription rate
δ	1.5	Transcript degradation rate
ε_A	2	free energy of polymerase binding*
F_{TF}	0.7 / 1 / 1.4	Regulation factor of the TF-network

Parameter set {Y} of the transcription machinery. Rates are given in events per simulation step. *scaled by the Boltzmann unit.

**Parameter**	**Value**	**Description**

$D_{main,0}$	0.99	Maximum probability of maintaining DNA methylation
$D_{novo,0}$	0.1	Maximum probability of *de novo* DNA methylation
D^{4}_{CG}, D^{27}_{CG}	6, 4	Interaction energy between HMTs and DNMTs*
E_{m1}, E_{m2}	2, 10	Energy constants describing accessibility for and binding of DNMT1 to methylated CpGs, respectively*

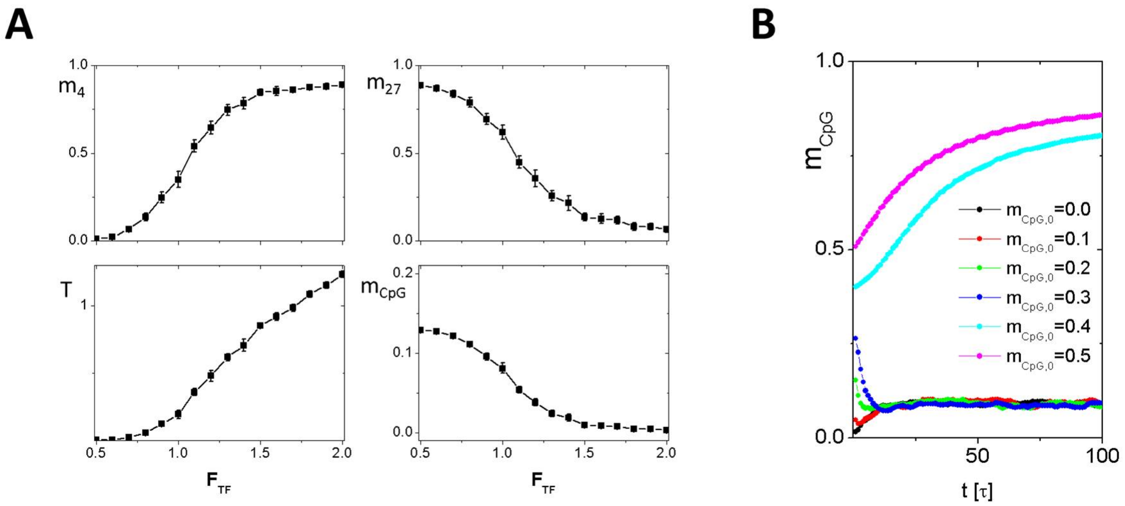

Parameter set {Z} of the DNA methylation machinery. Rates are given in events per simulation step. *scaled by the Boltzmann unit.

III. Basic properties of model genes

The regulatory states of the G1 model gene depend on its integration in the TF-network. As expected, increased transcriptional activation of G1, modelled increasing the regulation factor F_{TF}, increases its transcription and H3K4me3 level, while the H3K27me3 and DNA methylation levels decrease (Fig. S4A). Thereby, the regulatory states in individual cells fluctuate and can considerably deviate from population averaged values. These fluctuations nearly vanish if the genes adopts a high DNA methylation state. The G1 model gene approaches such a state if its DNA methylation, due to fluctuations or external regulation, reaches values above $m_{CpG,C}$ (for parameter set A: $0.3<m_{CpG,C}<0.4$, Fig. S4B).

Fig. S4: Equilibrium states of the G1 model gene. A) The regulatory states depend on the regulation factor F_{TF}. Shown are the states of the model gene averaged over 100 cells. Errors: SD, B) Increasing the methylation level of the initial state $m_{CpG,0}$, the gene will reach in the long term run the unmodified state with high DNA methylation, starting above $m_{CpG,0}=0.3$.

IV. Details on promoter DNA methylation scenarios

Low methylation of G1 promoters might originate in changes of either the maintenance or the *de novo* DNA methylation activity. However, HCG promoter genes strongly enriched in G1 genes. Accordingly, their promoters show low methylation levels. For such genes, our

in silico model predicts a weak dependence of the methylation level on the activity of the maintenance DNA methyltransferase DNMT1 (Fig. S5A). Thus, we focused on changes of the *de novo* DNA methyltransferases DNMT3a/b; changing the *de novo* DNA methylation activity D_{novo}.

Alternatively, low methylation might originate in activated hydroxyl-methylation [11,14]. In this scenario, G1 genes have to be targets of the ten eleven translocation (TET)-pathway. TET-proteins are often recruited by HCG promoters. Thus, we expected strong recruitment to G1 gene promoters in control mice, consistent with their low DNA methylation level. Utilizing published data [15] (E-MTAB-5202), we re-analyzed hydroxyl-methylation (5-hydroxymethylcytosine: 5hmC) in intestinal cells. In short, we calculated an average RPM value for the promoter of each gene using the processed RPM values as provided. A core promoter was defined as the 1kb broad region [TSS-500bp, TSS+500bp]. Thus, RPM and RPKM numbers are identically. Actually, we did not found an enrichment of 5hmc in G1 HCG compared to G2 LCG (ICG) promoters (Fig. S5B). So, low DNA methylation specifically at G1 promoters by improved TET recruitment is unlikely although we cannot exclude a contribution of this mechanism.

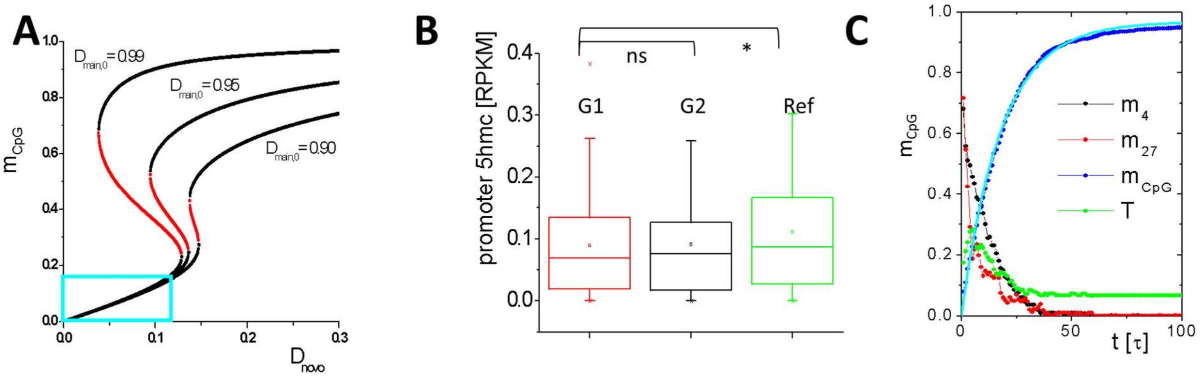

Fig. S5: Alternative de-methylation scenarios. A) In our *in silico* model, a decrease of the DNMT1 activity, modelled by decreasing $D_{main,0}$, strongly affects the level of methylation in high methylation states. In contrast, it has only a marginal effect on the level of methylation in low methylation states (cyan box). B) Box-plot of promoter 5hmc levels of selected gene sets in ISCs. The distribution of 5hmC levels for G1 genes is similar to G2 genes and shows a significantly lower mean than a subgroup of genes (Ref) that was predicted to be regulated by the TET-pathway during ISC specification and differentiation [16]. * $p<0.1$ (Kolmogorov-Smirnov-test) C) The simulated regulatory states depend on the maximum *de novo* methylation activity $D_{novo,0}$. For constant $D_{novo,0}=0.3> D_{novo,c}$, the probability of reaching the high methylation state (e') (compare Fig. 3A) approaches one. Starting from an H3K4me3- H3K27me3 state, the maximum of m_{CpG} is approached with an exponential decay time of 18τ (cyan line).

Simulating DNA repair, we temporary increased the maximum *de novo* DNA methylation activity $D_{novo,0}$. In case of a permanent increase to values above $D_{novo,C,}$, the G1 model gene definitely reaches the state of high DNA methylation (Fig. S5C).

V: Modelling increased chromatin accessibility of G1 genes.

Increasing the DNA binding strength of both H3K4me3 and H3K27me3 HTMs in parallel can be achieved by decreasing DNA methylation but also by increasing the chromatin accessibility. This can be modeled changing the parameters ε_0^k for both modifications (k=4, 27) in parallel (Supplementary II, Equation S1). We simulated the systems behavior

decreasing ε_0^k (k=4, 27) in identical steps Δε₀ = 0.1 (scaled by the Boltzmann unit). Simulation results for the regulatory states of the genes are provided in Fig. S6.

VI: Modelling promoter hyper-methylation during DNA repair.

Simulations of promoter DNA hyper-methylation are performed as described [9]. In contrast to this study, we here neglect changes of the regulatory factor F_{TF} due to transcriptional feedback via the TF-network. Moreover, we here provide properties of hyper-methylation of individual G1 model genes (average incidence of repair events before hyper-methylation, probability of hyper-methylation), and not rates of fixation of the methylation in the intestinal crypt following monoclonal conversion. In case of a random distribution of the repair events this would on average reduce the rate R to R/N_{ISC}, where N_{ISC} is the average number of ISCs in the crypt.

The properties are calculated starting at t=100τ, to minimize artefacts of particular high hyper-methylation rates of genes in state (a) (H3K27me3 only) after the onset of repair. Genes that are in state (a) have the highest probability to become hyper-methylated. Accordingly, the frequency of this state is reduced under DNA repair.

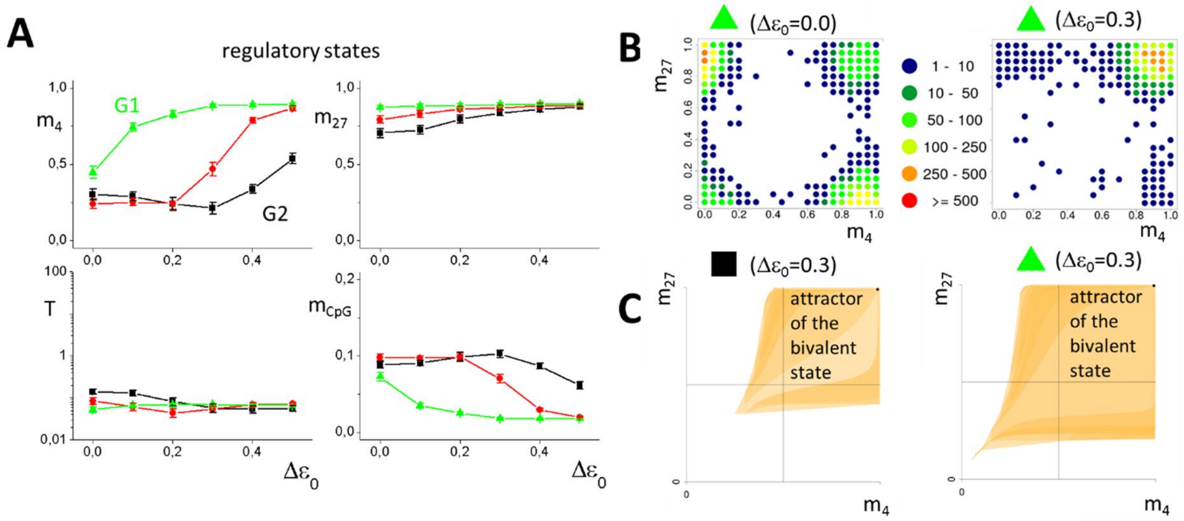

Fig. S6: Effects of increased chromatin accessibility. A) The regulatory states of the G1 model gene for increasing deviation $\Delta\varepsilon_0$ from the reference value of ground enthalpy of HMT complex binding ε_0 are shown. Similar to decreased DNA methylation, an immediate increase in H3K4me3 is seen for ε_{BS}=5.5 (green), while for lower values (5.2: red, 5.0: black) such an increase occurs for larger $\Delta\varepsilon_0$ only. Independent of ε_{BS} the transcription remains stable despite of H3K4me3 changes. In all simulations, H3K27me3 levels are high. Notably, the DNA methylation slightly decreases with increasing H3K4me3. B) Histograms of occupation frequency (total cell count: 5000) of the (H3K4me3, H3K27me3) states for ε_{BS}=5.5. At ε_0=0 a broad state distribution is seen (left), while at ε_0=0.3 the population becomes homogeneous and the G1 model gene is bivalent in most of the cells (right). C) Changes of the occupation frequency with ε_{BS} (5.0 (left), 5.5 (right), ε_0=0.3) originate in a changed size of the attractor of the bivalent state (beige area).

Reference

1. Herberg, M.; Siebert, S.; Quaas, M.; Thalheim, T.; Rother, K.; Hussong, M.; Altmüller, J.; Kerner, C.; Galle, J.; Schweiger, M.R.; Aust, G. Loss of Msh2 and a single-radiation hit induce common, genome-wide, and persistent epigenetic changes in the intestine. *Clin. Epigenetics* **2019**, *11*, 65, doi:10.1186/s13148-019-0639-8.

2. Kucherlapati, M.H.; Lee, K.; Nguyen, A.A.; Clark, A.B.; Hou, H., Jr.; Rosulek, A.; Li, H.; Yang, K.; Fan, K.; Lipkin, M.; et al. An Msh2 conditional knockout mouse for studying intestinal cancer and testing anticancer agents. *Gastroenterology* **2010**, *138*, 993–1002.
3. Martin, M. Cutadapt removes adapter sequences from high-throughput sequencing reads. *Embnet J.* **2011**, *17*, 10–12, doi:10.14806/ej.17.1.200. 17.
4. Andrews, S. FastQC: A Quality Control Tool for High Throughput Sequence Data. 2011. Available online: http://www.bioinformatics.babraham.ac.uk/projects/fastqc (accessed on 3 March 2020).
5. Hoffmann, S.; Otto, C.; Kurtz, S.; Sharma, C.M.; Khaitovich, P.; Vogel, J.; Stadler, P.F.; Hackermüller, J. Fast mapping of short sequences with mismatches, insertions and deletions using index structures. *PLoS Comput. Biol.* **2009**, *5*, e1000502, doi:10.1371/journal.pcbi.1000502.
6. Feng, J.; Liu, T.; Qin, B.; Zhang, Y.; Liu, X.S. Identifying ChIP-seq enrichment using MACS. *Nat. Protoc.* **2012**, *7*, 1728–1740.
7. Brown, G. GreyListChIP: Grey Lists -- Mask Artefact Regions Based on ChIP Inputs. 2019. R package version 1.18.0
8. Loeffler-Wirth, H.; Kalcher, M.; Binder, H. oposSOM: R-package for high-dimensional portraying of genome-wide expression landscapes on Bioconductor. *Bioinformatics* **2015**, *31*, 3225–3227.
9. Thalheim, T.; Herberg, M.; Galle, J. Linking DNA damage and age-related promoter DNA hyper-methylation in the intestine. *Genes* **2018**, *9*, 17, doi:10.3390/genes9010017.
10. Zhang, T.; Cooper, S.; Brockdorff, N. The interplay of histone modifications—writers that read. *EMBO Rep.* **2015**, *16*, 1467–1481.
11. Thalheim, T.; Herberg, M.; Loeffler, M.; Galle, J. The regulatory capacity of bivalent genes-a theoretical approach. *Int. J. Mol. Sci.* **2017**, *18*, 1069, doi:10.3390/ijms18051069.
12. Binder, H.; Wirth, H.; Galle, J. Gene expression density profiles characterize modes of genomic regulation: Theory and experiment. *J. Biotechnol.* **2010**, *149*, 98–114.
13. Binder, H.; Steiner, L.; Przybilla, J.; Rohlf, T.; Prohaska, S.; Galle, J. Transcriptional regulation by histone modifications: Towards a theory of chromatin re-organization during stem cell differentiation. *Phys. Biol.* **2013**, *10*, 026006, doi:10.1088/1478-3975/10/2/026006/meta.
14. Rasmussen, K.D.; Helin, K. Role of TET enzymes in DNA methylation, development, and cancer. *Genes Dev.* **2016**, *30*, 733–750.
15. Kim, R.; Sheaffer, K.L.; Choi, I.; Won, K.J.; Kaestner, K.H. Epigenetic regulation of intestinal stem cells by TET1-mediated DNA hydroxymethylation. *Genes Dev.* **2016**, *30*, 2433–2442.
16. Thalheim, T.; Hopp, L.; Binder, H.; Aust, G.; Galle, J. On the cooperation between epigenetics and transcription factor networks in the specification of tissue stem cells. *Epigenomes* **2018**, *2*, 20, doi:10.3390/epigenomes2040020.
